# Negative Symptom Domains Are Associated With Verbal Learning in Adolescents With Early Onset Psychosis

**DOI:** 10.3389/fpsyt.2021.825681

**Published:** 2022-01-07

**Authors:** Lynn Mørch-Johnsen, Runar Elle Smelror, Dimitrios Andreou, Claudia Barth, Cecilie Johannessen, Kirsten Wedervang-Resell, Laura A. Wortinger, Ricardo Díaz, Gamaliel Victoria, Torill Ueland, Ole A. Andreassen, Anne M. Myhre, Bjørn Rishovd Rund, Rosa Elena Ulloa, Ingrid Agartz

**Affiliations:** ^1^Norwegian Centre for Mental Disorders Research, Institute of Clinical Medicine, University of Oslo, Oslo, Norway; ^2^Department of Psychiatry and Department of Clinical Research, Østfold Hospital, Grålum, Norway; ^3^Department of Psychiatric Research, Diakonhjemmet Hospital, Oslo, Norway; ^4^Department of Clinical Neuroscience, Centre for Psychiatry Research, Karolinska Institutet and Stockholm Health Care Services, Stockholm County Council, Stockholm, Sweden; ^5^Department of Neurohabilitation, Oslo University Hospital Ullevål, Oslo, Norway; ^6^Research Department, Arete Proyectos y Administración, Mexico City, Mexico; ^7^Planning of Prevention Programs in the Directorate of Integral Attention to Girls, Boys and Adolescents, System for the Integral Development of the Family, Mexico City, Mexico; ^8^Division of Mental Health and Addiction, Norwegian Centre for Mental Disorders Research, Oslo University Hospital, Oslo, Norway; ^9^Department of Psychology, University of Oslo, Oslo, Norway; ^10^Institute of Clinical Medicine, University of Oslo, Oslo, Norway; ^11^Research Department, Vestre Viken Hospital Trust, Drammen, Norway; ^12^Developmental Psychopharmacology at the Research Division, Child Psychiatric Hospital, Mexico City, Mexico; ^13^Institute of Clinical Medicine, K.G. Jebsen Centre for Neurodevelopmental Disorders, University of Oslo, Oslo, Norway

**Keywords:** apathy, diminished expression, early-onset schizophrenia, MATRICS, MCCB, factor analysis

## Abstract

**Background:** Early-onset psychosis (EOP) is among the leading causes of disease burden in adolescents. Negative symptoms and cognitive deficits predicts poorer functional outcome. A better understanding of the association between negative symptoms and cognitive impairment may inform theories on underlying mechanisms and elucidate targets for development of new treatments. Two domains of negative symptoms have been described in adult patients with schizophrenia: *apathy* and *diminished expression*, however, the factorial structure of negative symptoms has not been investigated in EOP. We aimed to explore the factorial structure of negative symptoms and investigate associations between cognitive performance and negative symptom domains in adolescents with EOP. We hypothesized that (1) two negative symptom factors would be identifiable, and that (2) diminished expression would be more strongly associated with cognitive performance, similar to adult psychosis patients.

**Methods:** Adolescent patients with non-affective EOP (*n* = 169) were included from three cohorts: Youth-TOP, Norway (*n* = 45), Early-Onset Study, Norway (*n* = 27) and Adolescent Schizophrenia Study, Mexico (*n* = 97). An exploratory factor analysis was performed to investigate the underlying structure of negative symptoms (measured with the Positive and Negative Syndrome Scale (PANSS)). Factor-models were further assessed using confirmatory factor analyses. Associations between negative symptom domains and six cognitive domains were assessed using multiple linear regression models controlling for age, sex and cohort. The neurocognitive domains from the MATRICS Consensus Cognitive Battery included: speed of processing, attention, working memory, verbal learning, visual learning, and reasoning and problem solving.

**Results:** The exploratory factor analysis of PANSS negative symptoms suggested retaining only a single factor, but a forced two factor solution corroborated previously described factors of apathy and diminished expression in adult-onset schizophrenia. Results from confirmatory factor analysis indicated a better fit for the two-factor model than for the one-factor model. For both negative symptom domains, negative symptom scores were inversely associated with verbal learning scores.

**Conclusion:** The results support the presence of two domains of negative symptoms in EOP; apathy and diminished expression. Future studies on negative symptoms in EOP should examine putative differential effects of these symptom domains. For both domains, negative symptom scores were significantly inversely associated with verbal learning.

## Introduction

Early-onset psychosis (EOP) is defined as the onset of a psychotic disorder before 18 years of age (1). Although EOP is rare [affecting about 0.05–0.5% of the general population (2–4)], it is among the leading causes of disease burden in adolescents (5). Negative symptoms are present in 37–50% of EOP at illness onset (6, 7), and offer a particular challenge concerning outcome and quality of life as they are associated with poor functional outcome (8), cognitive impairments (9), and multiple treatment failures (6).

Negative symptoms commonly refer to symptoms reflecting diminished normal functions and behaviors, including alogia, blunted affect, anhedonia, asociality, and avolition (10). Studies investigating the factorial structure of different negative symptom rating scales in adult-onset schizophrenia, suggest that negative symptoms consist of two or more factors (11, 12). Most consistently reported and investigated are the two domains: *apathy*, including avolition, asociality and anhedonia, and *diminished expression*, including blunted affect and alogia (11, 13–16). Previous studies examining the factorial structure of negative symptoms in the widely used Positive and Negative Syndrome Scale [PANSS; (17)] have confirmed the two domains, and the models have been largely convergent (13, 14, 18). The reported two-factor structure comprise of: (1) an *apathy* domain, including emotional withdrawal, passive social withdrawal, and active social avoidance and (2) a *diminished expression* domain, including blunted affect, poor rapport, lack of spontaneity, and motor retardation (13, 14). This structure has been supported by a confirmatory factor analysis in an adult schizophrenia sample (19), and validated against corresponding subdomains of the Brief Negative Symptom Assessment Scale (20). Investigations of the two negative symptom domains, separately, have reported differential associations with other clinical aspects of psychotic disorders and neurobiology, including functional outcome (21), cognitive impairments (22, 23), neuronal task activation (24), and white matter connectivity (25). Although the exact mechanisms still need to be elucidated, these results may indicate different underlying pathophysiology (26), which may require different treatment approaches. Current conceptualizations of negative symptoms advocate the importance of deconstructing this symptom construct into separate symptoms and dimensions to achieve a better understanding of the phenomenology, and the functional and biological correlates (15, 27).

In adult-onset schizophrenia, associations with cognitive deficits have been shown for both apathy and diminished expression when the domains have been investigated separately (26). Specific problems with executive functioning and working memory may be associated with motivational deficits and reduced goal directed behavior in the apathy domain (26). More general cognitive impairments, according to the “cognitive resource limitation model”, have been proposed to contribute to diminished expression symptoms (26, 28, 29). Some studies exploring the putative associations between cognition and the two negative symptom domains, have suggested a stronger association to cognitive impairments for diminished expression, than for apathy (22, 23, 30).

Adolescence is a sensitive developmental period associated with rapid neuro-maturational changes (31). Negative symptoms and cognitive difficulties are particularly challenging as currently available treatment is not adequately effective (32, 33). Studies of children and adolescents with EOP have demonstrated higher genetic heritability, poorer premorbid adjustment, longer duration of untreated psychosis (DUP), more severe illness course and outcome, and higher suicide rate, relative to patients with adult-onset psychosis (7, 34–36). These findings illustrate a crucial need for increased knowledge of pathological mechanisms associated with EOP. In a previous study from our group including an overlapping EOP sample with the present study, negative and disorganized symptoms were found to mediate the relationship between verbal learning and global functioning (37). However, to the best of our knowledge, the factorial structure of negative symptoms and how specific subdomains of negative symptoms relate with cognition, have not yet been investigated in adolescent patients with EOP. A better understanding of the phenomenology of negative symptoms, and how these symptoms relate to cognitive domains may improve early detection and inform theories on underlying mechanisms.

Thus, we aimed to (1) explore the factorial structure of the negative symptom construct and (2) investigate associations between cognitive impairments and negative symptom domains in EOP. We hypothesized that two factors of negative symptoms will be identifiable; an apathy factor and a diminished expression factor, and that diminished expression is more strongly associated with cognitive impairments, in accordance with studies of adult patients [e.g., (22, 23)].

## Materials and Methods

### Participants

The subject sample included 169 adolescents with non-affective EOP with the following diagnoses: schizophrenia (*n* = 101), schizophreniform disorder (*n* = 33), schizoaffective disorder (*n* = 4), brief psychotic disorder (*n* = 2) and psychosis not otherwise specified (*n* = 29). Participants were recruited from three different cohorts: (1) the Thematically Organized Psychosis Study for Youth (Youth-TOP), Norway (*n* = 45, recruited from 2013 to 2019), (2) the Early-Onset Study, Norway (*n* = 27, recruited from 2005 to 2007) and (3) the Adolescent Schizophrenia Study, Mexico (*n* = 97, recruited from 2011 to 2020). The Norwegian cohorts were recruited from adolescent inpatient units and outpatient clinics in the south-east area of Norway (mainly the Oslo area). The Mexican cohort was recruited from an inpatient unit at the Child and Adolescent Psychiatric Hospital in Mexico City.

Inclusion criteria for the current study were: (1) A non-affective psychotic disorder, verified according to the Diagnostic and Statistical Manual of Mental Disorders, fourth edition (DSM-IV) (38), (2) age 12–18 years, and (3) adequate language abilities to complete the interviews and self-rating questionnaires. Patients were excluded if they had a substance-induced psychotic disorder, organic brain disease, previous moderate/severe head injury, or IQ outside of the normal range. IQ was formally tested in the participants from the Youth-TOP and Early-Onset Study using the Wechsler Abbreviated Scale of Intelligence (39), and participants with IQ below 70 were excluded. In the Adolescent Schizophrenia Study, IQ was considered within the normal range if the patient did not have significant developmental delays and was attending regular school without any formal educational support.

All participants (and/or legal guardians if age <16 years) were thoroughly informed about the study and signed a written consent form. The Youth-TOP and Early-Onset Study were approved by the Norwegian South-East Regional Committee for Medical Research Ethics and the Norwegian Data Inspectorate. The Adolescent Schizophrenia Study was approved by the Child Psychiatric Hospital Ethics Committee. All studies were conducted in accordance with the Helsinki declaration.

### Clinical Assessments

#### Diagnosis and Global Functioning

Diagnoses were established according to the DSM-IV, using the following structured interviews: (1) Youth-TOP study: the Schedule for Affective Disorders and Schizophrenia for School Aged Children – Present and Lifetime Version (K-SADS-PL) (40), (2) Early-Onset Study: the Structural Clinical Instrument of Diagnosis for DSM-IV Axis I disorders (SCID-I), module A-D (41), and (3) Adolescent Schizophrenia Study: the Mini International Neuropsychiatric Interview (MINI-KID) (42).

Global functioning was assessed using three different scales: the Youth-TOP study: the Children's Global Assessment Scale (CGAS) (43), (2) Early-Onset Study: the Global Assessment of Functioning Scale, split version (GAF-F) (44), (3) Adolescent Schizophrenia Study: the Personal and Social Performance Scale (PSP) (45).

#### Negative Symptoms

Characteristic symptoms of psychosis, including negative symptoms were assessed using the PANSS (17). Although the PANSS was originally developed for adults, it has been used in several studies in adolescent patients (46, 47). In line with previous work in adult patients with schizophrenia (13, 14), negative symptom items from the negative symptom factor scores published by Marder and colleagues (48) were included for the exploratory factor analysis. The PANSS items included: n1 (blunted affect), n2 (emotional withdrawal), n3 (poor rapport), n4 (passive/apathetic social withdrawal), n6 (reduced spontaneity and flow of conversation), g7 (motor retardation), g16 (active social avoidance).

#### Cognitive Measures

Six cognitive domains were investigated based on nine tests from the MATRICS Consensus Cognitive Battery (MCCB) (49): (1) *Speed of processing* [combining BACS Symbol coding (50), Trail making test, part A (51), and Category fluency (52)], (2) *Attention/vigilance* (Continuous performance test, identical pairs) (53), (3) *Working memory* [combining WMS-III Spatial span (54) and Letter-number span (55)], (4) *Verbal learning* (Hopkins verbal learning test, revised) (56), (5) *Visual learning* (Brief visuospatial memory test, revised) (57), and (6) *Reasoning and problem solving* (NAB Mazes) (58). Although the MCCB was developed for adult patients with schizophrenia, the cognitive tests have been successfully used in adolescent EOP patients (59–62) and healthy adolescents (63, 64). The social cognition test (MSCEIT: Managing emotions) (65) included in the MCCB has been shown to be less suitable for adolescents (61) and was therefore excluded from the analyses in the current study. A global composite cognition score was calculated based on the nine included tests.

#### Medication

Information on current use of psychotropic medication was assessed, and chlorpromazine (CPZ) equivalents (66) were calculated for antipsychotic medication.

### Statistical Analyses

Statistical analyses were performed with SPSS (version 27), except for the confirmatory factor analysis which was performed using R (version 4.0.5).

#### Clinical and Demographic Data

Demographic and clinical data were compared between cohorts using analysis of variance (ANOVA) and chi-square statistics. All tests were two-tailed.

#### Factor Analysis

The exploratory factor analysis (EFA, Principal Axis Factoring, SPSS) was performed to investigate the underlying structure of negative symptoms. The Kaiser-Meyer-Oklin (KMO) and Bartlett's test of sphericity were calculated to assess sampling adequacy. Number of factors to retain was determined based on Kaiser's criterion of an eigenvalue >1, and visual inspection of the scree plot. As two factors have been demonstrated in adult schizophrenia populations (13, 14, 18) we also explored setting the number of factors to be retained to two. The Promax oblique rotation was applied as we expected factors to be correlated, and items with loadings >0.3 were used for factor interpretation.

To further assess the models derived from the exploratory factor analysis, confirmatory factor analysis was conducted using the Lavaan package in R (67). Because of non-normality of data, the maximum likelihood estimation with robust standard errors and Satorra-Bentler scaled test statistics was used (68). Two models were assessed: (1) a one-factor model (n1, n2, n3, n4, n6, g7 and g16), and (2) the two-factor model of diminished expression (n1, n3, n6 and g7) and apathy (n2, n4 and g16) (13, 14). Goodness-of-fit was evaluated using different indices: chi-square, comparative fit index (CFI >0.95), Tucker-Lewis index (TLI >0.95), root mean square error of approximation (RMSEA <0.06), and standardized root mean square residual (SRMR <0.08) (69). As chi-square may be affected by sample size, a normed chi-square was calculated by dividing the chi-square by degrees of freedom, and a value below 5.0 was considered acceptable (70).

#### Associations of Negative Symptom Domains and Cognition

Based on previously published negative symptom factor models in adult-onset schizophrenia using PANSS (13, 14), scores for avolition-apathy (n1 + n3 + n6 + g7) and diminished expression (n2 + n4 + n6) were calculated by summing the scores of items included in each factor. Putative associations between cognitive domains and negative symptom factors were investigated using separate multiple linear regression models, controlling for age, sex, and cohort. Sex-specific associations were assessed by exploring models including sex-by-negative domain interactions. The linear regression models were investigated for influential cases. Standardized residuals >3 were identified for the models in speed of processing (3 cases), working memory (1 case) and global cognition (2 cases). However, Cook's distances did not exceed a value of 1 and all cases were retained in the analyses. The cognitive raw tests scores from the MCCB were transformed to standard scores (Z scores) using the standardization function in SPSS. For composite scores such as speed of processing, working memory and global cognition, Z scores from the individual tests were summated and transformed into a composite Z score, in line with the recommended procedure from the MCCB standardization study (71). The TMT-A score included in the speed of processing domain was reversed as high scores on this test indicate lower performance.

As the cognitive domains are not independent, a modified Bonferroni correction that accounts for correlations between outcome variables was used (72). Applying this method resulted in a *p*-value threshold of *p* < 0.018 (accounting for 7 domains and an intra-class correlation coefficient of 0.714).

Significant associations between cognition and negative symptom domains were investigated for the influence of PANSS positive and depressive symptom factors (73) and antipsychotic medication (antipsychotic medication use, and among patient using antipsychotics; CPZ equivalents).

## Results

### Clinical and Demographic Data

Demographic and clinical data for the patients are presented in Table 1, and comparisons on mean scores on cognitive domains in Table 2. The Adolescent Schizophrenia Study consisted of significantly more males, with greater symptom severity, and lower cognitive performance compared to the Youth-TOP and the Early-Onset study. Patients in the Youth-TOP study had more PANSS negative and disorganized symptoms compared to the Early-Onset study. Use of antipsychotic medication was more prevalent in the Adolescent Schizophrenia Study, but there were no significant differences in medication dose (CPZ-equivalents) between the cohorts.

**Table 1:** Demographic and clinical data.

	**All** ***n*** **= 169**		**1. Youth-TOP** ***n*** **= 45**		**2.Early-Onset Study** **n** **= 27**		**3.Adolescent Schizophrenia Study** ***n*** **= 97**			
	**Mean/n**	**SD/%**	**Mean/n**	**SD/%**	**Mean/n**	**SD/%**	**Mean/n**	**SD/%**	**Test statistics**	***Post-hoc***
Diagnoses									χ^2^ = 51.30, *p* < 0.001	
Schizophrenia spectrum	138	81.7%	25	55.6%	16	59.3%	97	100%		
Other psychosis^a^	31	18.2%	20	44.4%	11	40.7%	0	0		
Age	15.5	1.5	15.6	1.3	15.9	1.8	15.4	1.6	F = 1.42, *p* = 0.24	
Sex, male	100	59.2%	16	35.6%	13	48.1%	71	73.2%	χ^2^ = 19.64, *p* < 0.001	3>1, 3>2
Hand dominance, right	156	92.3%	42	93.3%	23	85.2%	91	93.8%	χ^2^ = 2.30, *p* = 0.316	
Ethnicity									χ^2^ = 163.14, *p* < 0.001	
Caucasian	59	34.7%	38	84.4%	21	77.8%	0	0		
Hispanic	97	57.6%	1	2.2%	0	0	96	99.0%		
Other	13	7.6%	6	13.3%	6	22.6%	1	1.0%		
Age of onset	14.0	1.9	14.2	1.7	14.1	2.0	13.9	1.9	F = 0.42, *p* = 0.659	
Global functioning^b^			45.2	11.7	48.0	15.2	34.6	13.1		
PANSS positive^c^	14.3	5.1	11.3	3.6	9.8	3.3	16.9	4.4	F = 49.78, *p* < 0.001	3>1, 3>2
PANSS negative^c^	18.5	7.6	16.6	6.3	11.6	4.5	21.2	7.4	F = 23.84, *p* < 001	1>2, 3>1, 3>2
PANSS disorganized^c^	9.0	4.1	6.7	2.6	4.8	1.9	11.2	3.5	F=63.16, p <0.001	1>2, 3>1, 3>2
PANSS depression^c^	7.6	3.3	8.4	2.9	7.8	3.4	7.2	3.4	F = 1.85, *p* = 0.161	
PANSS excited^c^	9.1	4.4	6.6	2.0	6.7	2.5	11.0	4.7	F = 26.71, *p* < 0.001	3>1, 3>2
PANSS total	85.9	25.7	71.6	15.2	57.5	12.9	100.5	21.5	F = 72.53, *p* < 0.001	1>2, 3>1, 3>2
Apathy	3.3	1.3	3.0	1.1	2.0	0.8	3.8	1.2	F = 32.08, *p* < 0.001	1>2, 3>1, 3>2
Diminished expression	2.9	1.3	2.6	1.1	1.9	0.9	3.3	1.3	F = 15.79, *p* < 0.001	1>2, 3>1, 3>2
Antipsychotic use	144	85.2%	28	62.2%	19	70%	97	100%	χ^2^ = 40.41, *p* < 0.001	3>1, 3>2
Antipsychotic dose (CPZ^d^)	245.0	128.4	236.3	137.0	263.5	181.0	243.9	116.6	F = 0.26, *p* = 0.772	

a *Other psychosis: Brief psychotic disorder (n = 2), psychosis not otherwise specified (NOS, n = 29).*

b *Global functioning: Children's Global Assessment Scale (Youth-TOP), Global Assessment of Functioning Scale (Early-Onset Study), and the Personal and Social Performance Scale (Adolescent Schizophrenia Study).*

c *Positive and Negative Syndrome Scale Wallwork 5-factor model.*

d *Chlorpromazine equivalents*.

**Table 2:** Cognition across cohorts.

	**All**		**1. Youth-TOP**		**2.Early-Onset Study**		**3.Adolescent Schizophrenia Study**		**F**	**p**	***Post-hoc*, *p* < 0.05**
**Domain**	**Mean Z score**	**SD**	**Mean Z score**	**SD**	**Mean Z score**	**SD**	**Mean Z score**	**SD**			
Speed of processing	−0.11	1.00	0.55	0.63	0.34	0.59	−0.50	1.03	23.8	<0.001	3>1, 3>2
Attention/vigilance	−0.08	0.96	0.47	0.91	0.28	0.92	−0.43	0.84	18.0	<0.001	3>1, 3>2
Working memory	−0.09	0.97	0.58	0.77	0.25	0.61	−0.51	0.93	28.4	<0.001	3>1, 3>2
Verbal learning	−0.05	1.00	0.51	0.82	0.21	0.84	−0.38	0.99	15.4	<0.001	3>1, 3>2
Visual learning	−0.05	1.02	0.52	0.81	0.26	1.05	−0.41	0.95	17.1	<0.001	3>1, 3>2
Reasoning and problem solving	−0.11	0.98	0.64	0.76	0.41	0.80	−0.60	0.82	44.3	<0.001	3>1, 3>2
Global cognition	−0.10	0.96	0.71	0.67	0.35	0.58	−0.55	0.92	35.2	<0.001	3>1, 3>2

### Factor Analysis

The exploratory factor analysis showed excellent Kaiser-Meyer-Oklin value of 0.886, and a significant Bartlett's test (*p* < 0.001), indicating adequate sample size and correlation matrix for factor analysis. When considering both criteria of eigenvalue >1 and visual inspection of the scree plot, one factor was retained (eigenvalue of 4.758, explaining 68% of the variance). As shown in Table 3, when the model was forced to extract two factors, items n2, n4 and g16 loaded highly on factor 1 (corresponding to the avolition-apathy domain) and n1, n3, n6 and g7 loaded highly on factor 2 (corresponding to the diminished expression domain. Factors 1 and 2 were highly correlated 0.798. The two domains showed good internal consistency as demonstrated by a Cronbach's alpha of 0.852 for the apathy domain, and 0.890 for the diminished expression domain.

**Table 3:** Factor structure.

**PANSS item**	**Factor 1** **Apathy**	**Factor 2** **Diminished expression**
N1 Blunted affect		**0.595**
N2 Emotional withdrawal	**0.902**	
N3 Poor rapport	0.418	**0.498**
N4 Passive/apathetic social withdrawal	**0.891**	
N6 Lack of spontaneity		**0.795**
G7 Motor retardation		**0.794**
G16 Active social avoidance	**0.569**	

*Pattern coefficients from exploratory factor analysis on PANSS items N1, N2, N3, N4, N6, G7, G16, forced two factor solution, after Promax rotation. For simplicity, only item loadings >0.3 are shown. Bolded values indicate the factor with the strongest loading*.

Results from the confirmatory factor analysis for the one- and two-factor models are presented in Table 4. Overall, goodness-of-fit statistics were better for a two-factor model than for a one-factor model, with a smaller chi-square as compared to the one-factor model, and values for normed chi-square (2.84), CFI (0.964), and SRMR (0.033) indicating a good fit.

**Table 4:** Results from confirmatory factor analysis.

	**One-factor model**	**Two-factor model**
Chi-square	62.210 (*p* < 0.001)	36.938 (*p* < 0.001)
Normed chi-square	**4.44**	**2.84**
CFI	0.928	**0.964**
TLI	0.892	0.942
RMSEA	0.160	0.117
SRMR	**0.045**	**0.033**

*CFI, comparative fit index (CFI > 0.95); TLI, Tucker-Lewis index (TLI > 0.95); RMSEA root mean square error of approximation (RMSEA < 0.06). SRMR, standardized root mean square residual (SRMR <0.08). Values within the considered thresholds for adequate fit are bolded*.

### Associations of Negative Symptom Domains and Cognition

Negative symptom scores for both apathy (β = −0.257, *p* = 0.002) and diminished expression (β = −0.259, *p* = 0.001) were inversely associated with verbal learning scores. An association was also seen between diminished expression and speed of processing (β = −0.173, *p* = 0.024), but this result was not significant after correction for multiple comparisons. No other significant associations were observed between the negative symptom domains and cognitive performance (Table 5). There were no significant sex-by-negative domain interactions.

**Table 5:** Associations between negative symptom domains and cognition.

	**Apathy**					**Diminished expression**				
	**B**	**SE**	**β**	***t***	***p***	**B**	**SE**	**β**	***t***	***p***
Speed of processing, *n* = 164	−0.122	0.063	−0.159	−1.932	0.055	−0.136	0.060	−0.173	−2.279	0.024^*^
Attention/vigilance, *n* = 157	0.029	0.065	0.038	0.440	0.660	−0.008	0.063	−0.011	−0.133	0.894
Working memory, *n* = 165	0.029	0.061	0.037	0.472	0.637	−0.018	0.059	−0.023	−0.307	0.759
Verbal learning, *n* = 168	−0.197	0.064	−0.257	−3.090	0.002^**^	−0.204	0.061	−0.259	−3.368	0.001^**^
Visual learning, *n* = 169	−0.010	0.067	−0.012	−0.146	0.884	−0.056	0.063	−0.070	−0.889	0.375
Reasoning and problem solving, *n* = 170	−0.018	0.056	−0.024	−0.323	0.747	−0.036	0.054	−0.047	−0.673	0.502
Global cognition, *n* = 148	−0.006	0.063	−0.008	−0.099	0.921	−0.081	0.061	−0.101	−1.324	0.188

*Results from the separate linear regression models controlled for age, sex and cohort.*

* *Significant at p < 0.05*

** *Significant after correction for multiple comparisons*.

The association between verbal learning and the two negative symptom domains remained significant after controlling for positive psychotic symptoms, depressive symptoms and antipsychotic medication use and dose.

## Discussion

In the present study, we explored the factorial structure of negative symptoms in patients with EOP and investigated how domains of negative symptoms were related to cognition. Overall, our results indicated a factorial structure of two domains similar to what has been shown for PANSS negative symptoms in adults (13, 14). However, the two factors were highly correlated. Both negative symptom domains were significantly associated with verbal learning.

We performed an exploratory factor analysis to investigate the factorial structure of negative symptoms in EOP as to our knowledge, this has not been investigated in EOP before. When considering standard criteria for factor retention, such as retaining only factors with an eigenvalue above 1 or by investigating the scree plot, the results suggested that only one factor should be retained. However, because of the theoretically supported model of two factors from adult schizophrenia, we explored forcing the extraction of two factors. Interestingly, the pattern of item loadings that emerged were identical to the two-factor model described from factor analytic studies in adult patients with schizophrenia (13, 14). PANSS items addressing blunted affect, poor rapport, lack of spontaneity, and motor retardation loaded the highest on a factor corresponding to a *diminished expression* domain, and emotional withdrawal, passive social withdrawal, and active social avoidance loaded highest on a second factor corresponding to an *apathy* domain. We further assessed both a one-factor model and a two-factor model (13, 14) using confirmatory factor analysis. Goodness-of-fit indices were better for the two-factor model, supporting this latent structure of two domains of negative symptoms in EOP. Discrepancies in the results from the exploratory and confirmatory factor analyses may reflect the different methodology and rationale for the two methods. In the exploratory factor analysis, the number of factors are explored in a data-driven approach, while the confirmatory factor analysis tests a predefined factor-model. Our sample size, although large with respect to EOP studies, did not allow for splitting the sample to perform exploratory and confirmatory factor analyses in separate samples. As such, replications of the confirmatory factor analysis should be performed in independent samples for generalizability.

An important implication of investigating subdomains of negative symptoms is that if such subdomains exist, they may have different biological and clinical correlates and may require different treatment strategies (15, 26). For instance, for the apathy domain, behavioral and neural dysfunctions related to motivation and goal-directed behavior have been shown, which have inspired the emerging research on targeted treatment options (26). Generally, models for underlying mechanisms of diminished expression are less clear (15, 26). One line of research points to cognitive deficits underlying symptoms of this domain (26, 28, 29). In support of this theory, a stronger association to cognitive deficits for diminished expression than apathy has been shown in some studies of adult patients with schizophrenia, although the overall differences are not large, and not consistent regarding the cognitive domains involved (22, 23, 30). In the present study of adolescents with EOP, apathy and diminished expression were similarly associated with lower verbal learning performance.

Overall, our results show that, although the factor analysis supports two domains of negative symptoms, these two domains could not be as clearly discriminated in our sample of patients with EOP as has been shown in previous studies of adult patients with schizophrenia (13, 14, 18). This may reflect differences between patients with adult- and early-onset schizophrenia. EOP has been associated with higher genetic heritability, poorer premorbid adjustment, longer duration of untreated psychosis (DUP), more severe illness course and outcome, and higher suicide rate, relative to patients with adult-onset psychosis (7, 34–36). Furthermore, the clinical presentation of underlying pathology may be different in adolescents who are in a period of life where the brain is rapidly changing, and cognitive abilities are developing.

Verbal learning deficits have been associated with earlier age of onset (74), and shown to be one of the earliest predictors of psychosis development in at-risk individuals (75, 76). Thus, verbal learning deficits and negative symptoms may be early markers for psychosis development and functional decline in youth. As cognitive assessment is better at predicting psychosis development in adolescents than in adults (77), our results indicate that clinicians working with young people need to be attentive to both verbal learning difficulties and negative symptoms. The patients presenting with these symptoms may represent a subgroup who may require closer follow-up and quick access to alternative treatment strategies in addition to antipsychotic medication, such as cognitive remediation. Furthermore, the results encourage future studies on how verbal learning and negative symptoms are associated and whether they relate to common underlying neurobiology.

Strengths of the study include a large and well-characterized sample of patients with early onset psychosis, which allowed for performing a factor analysis. Furthermore, a complete and standardized battery (MCCB) for cognitive testing was used. Nevertheless, some limitations should be mentioned. First, as a manifest diagnosis of a psychotic disorder is relatively rare in adolescents, combining samples from geographically different cohorts was necessary to obtain a sufficient sample size, however this may introduce unwanted variation related to cohort. There were significant cohort differences in sex, symptom severity and cognitive scores between cohorts. To address this concern all our multivariate analyses were controlled for cohort. Second, there are limitations to the PANSS as an assessment of negative symptoms (12). The ratings of negative symptoms in PANSS are based only on observation of behaviors, and not the subjective experience of the patients. For symptoms within the domain of apathy, this means that the patient's own experience of pleasure and motivation is not assessed. Newer scales for assessment of negative symptoms have been developed, so called “second-generation rating scales” (12), for instance the Brief Negative Symptom Scale (BNSS) (78) and the Clinical Assessment Interview for Negative Symptoms (CAINS) (79)) have been developed that include assessment of the subjective experience of symptoms within the apathy domain. However, these scales are currently not widely used in adolescents. Third, we included PANSS items for motor retardation (G7) and active social avoidance (G16) in the factor analysis, in line with previous factor analytic studies on negative symptoms in adult patients with schizophrenia. However, it should be noted, that recent guidelines from the European Psychiatric Association (12) on the assessment of negative symptoms advise against including these items as negative symptoms due to their inconsistent loading on the negative symptom factor. Fourth, high total PANSS scores indicate that some patients were in an acute or subacute phase of illness, which may have influenced their cognitive performance. Furthermore, positive symptoms, psychotropic medication, and depression may contribute to secondary negative symptoms. In our multivariate models on cognitive measures, we controlled for such possible secondary sources of negative symptoms (positive symptoms, depression and antipsychotic medication), but this did not change any results. Further, given the young age of the patients, we would expect them to be less influenced by chronicity and medication.

In conclusion, the results support the presence of two domains of negative symptoms in EOP, but the domains were highly correlated, and should be confirmed in independent samples. Contrary to our hypothesis of a stronger association between diminished expression and cognition, we found that for both domains, the negative symptom scores were similarly significantly associated with lower verbal learning scores. Based on the results, we recommend that future studies of negative symptoms in adolescents should examine differential effects of the two negative symptom domains. Furthermore, the association between negative symptoms and verbal learning warrants more studies on how these features are related and whether they for instance share common biological mechanisms that could be targeted for treatment.

## Data Availability Statement

The datasets presented in this article are not readily available because of legal and privacy restrictions. Requests to access the datasets should be directed to lynn.morch-johnsen@medisin.uio.no.

## Ethics Statement

The studies involving human participants were reviewed and The Youth-TOP and Early-Onset Study were approved by the Norwegian South-East Regional Committee for Medical Research Ethics and the Norwegian Data Inspectorate. The Adolescent Schizophrenia Study was approved by the Child Psychiatric Hospital Ethics Committee, Mexico. Written informed consent to participate in this study was provided by the participants' legal guardian/next of kin.

## Author Contributions

LM-J, RS, and IA took part in designing the analyses for present study. LM-J carried out the statistical analysis. LM-J and RS managed the literature search and wrote the first draft of the manuscript. RS, CJ, KW-R, RD, GV, AM, BR, RU, and IA were involved in data collection. All authors, LM-J, RS, DA, CB, CJ, KW-R, RD, GV, LW, TU, OA, AM, BR, RU, and IA have contributed to and approved the manuscript.

## Funding

This work was supported by the South-Eastern Norway Regional Health Authority (2019-108, 2019-099, 2004-259, 2006-186, 2020-020) and the Research Council of Norway (223273, 213700, 250358).

## Conflict of Interest

OA has received speaker's honorarium from Lundbeck and Sunovion and is a consultant for HealthLytix. The remaining authors declare that the research was conducted in the absence of any commercial or financial relationships that could be construed as a potential conflict of interest.

## Publisher's Note

All claims expressed in this article are solely those of the authors and do not necessarily represent those of their affiliated organizations, or those of the publisher, the editors and the reviewers. Any product that may be evaluated in this article, or claim that may be made by its manufacturer, is not guaranteed or endorsed by the publisher.
